# Hepatic Rupture Associated With HELLP (Hemolysis, Elevated Liver Enzymes, and Low Platelets) Syndrome: A Report of Two Cases and Literature Review

**DOI:** 10.7759/cureus.56627

**Published:** 2024-03-21

**Authors:** Homero Loza, Gabriela Carrión, Alexis Haro, Felipe Loza

**Affiliations:** 1 Obstetrics and Gynecology, Hospital de los Valles, Quito, ECU; 2 Obstetrics and Gynecology, Hospital General Dr. Enrique Ortega Moreira, Guayaquil, ECU; 3 Obstetrics and Gynecology, Universidad Espíritu Santo (UEES) Clinic, Guayaquil, ECU; 4 Obstetrics and Gynecology, Universidad Internacional del Ecuador, Quito, ECU; 5 Obstetrics and Gynecology, Axxis Hospital, Quito, ECU

**Keywords:** hepatic hematoma, pregnancy, liver hematoma, hellp syndrome, severe preeclampsia, hepatic rupture

## Abstract

Hepatic rupture is a rare complication of severe preeclampsia. A high index of suspicion is required in the presence of abdominal pain accompanied by hemodynamic decompensation in a pregnant woman. Hepatic rupture constitutes a medical emergency that demands immediate intervention, often with the support of other medical disciplines, in a highly specialized hospital setting. Unruptured hepatic hematomas can be managed conservatively. Immediate delivery and surgical repair of the liver are necessary for maternal survival. Spontaneous liver rupture in pregnancy is often unrecognized, highly lethal, and not completely understood with few cases having been reported in the literature. Therefore, we present two cases of HELLP (hemolysis, elevated liver enzymes, and low platelets) syndrome with hepatic rupture, emphasizing their clinical presentation and therapeutic approaches.

## Introduction

The HELLP (hemolysis, elevated liver enzymes, and low platelets) acronym was first used in 1982 by Weinstein to describe the syndrome of hemolysis, elevated liver function tests, and low platelets occurring in pregnancy as a severe form of preeclampsia [1]. Preeclampsia is a multisystem progressive disorder with systemic endotheliosis and vasospasm characterized by the new onset of hypertension and proteinuria or the new onset of hypertension plus significant end-organ dysfunction with or without proteinuria, typically presenting after 20 weeks of gestation or postpartum. However, it may present even without hypertension or proteinuria in 15-20% of the patients. HELLP syndrome may develop in 10-20% of them [2]. It affects 3-10% of all gestations and is responsible for 20-80% of deaths during pregnancy in low and middle-income countries; Fortunately, HELLP syndrome only affects 0.1-1% of pregnancies [3-5].

Hepatic rupture is a severe complication of HELLP syndrome and preeclampsia. A systematic review of 93 cases of hepatic rupture or hematoma published between 2000 and 2018 reported an elevated perinatal mortality of 44% and a maternal mortality of 7.5% [2]. This complication only occurs in 1 of 67,000 to 225,000 pregnancies [6-8].

Its pathophysiology is attributed to hepatic vascular congestion, secondary to periportal necrosis, fibrin deposition, and vasospasm: all of these being manifestations of placental failure. After the microcirculation ischemia/infarction, reperfusion circulation would cause a hepatic hemorrhage, subsequently forming a hematoma that could be ruptured [9].

This report describes the management experience of two cases of hepatic rupture secondary to severe HELLP syndrome and preeclampsia in 2021 in Ecuador.

## Case presentation

Case 1

**Figure 1:**
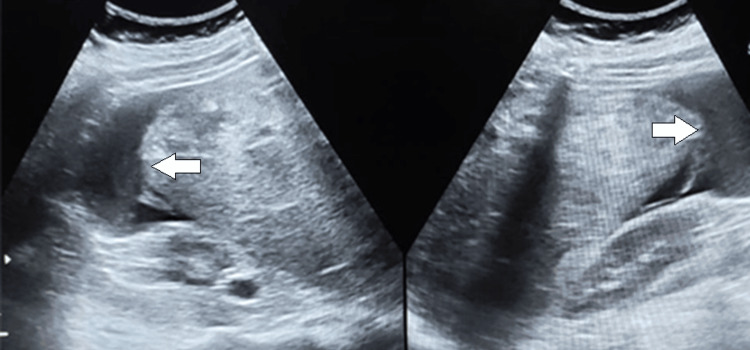
Ultrasonographic examination of the abdomen shows nonspecific heterogeneous hepatic parenchyma and perihepatic free fluid effusion (arrow).

A 38-year-old woman with an obstetric history of two successful cesarean deliveries (the first cesarean section was performed for presentation dystocia and the second because of the previous cesarean section) presents to the emergency department with a 32-week zero-day pregnancy and epigastric pain of 24 hours and five episodes of vomiting. Upon arrival at the emergency room, the vital signs were as follows: blood pressure 145/94, oxygen saturation 94%, temperature 36.9°C, and heart rate 92. An obstetric ultrasound revealed polyhydramnios and a living fetus with a heart rate of 151 beats per minute (bpm) with an omphalocele. The upper abdomen ultrasound showed a sub-hepatic hematoma on the right hepatic lobe (Figure 1).

Laboratory parameters at the presentation are mentioned in Table 1. Blood studies showed elevated liver enzymes, including alanine transaminase (ALT), aspartate transaminase (AST), alkaline phosphatase (ALP), and lactate dehydrogenase (LDH). A complete blood count showed leukocytosis with neutrophilia. No other values were abnormal. Mild hyperkalemia and high lactate were also present. Blood gas studies revealed metabolic acidosis with a blood pH of 7.20 (normal value is 7.35-7.45).

**Table 1:** Cases 1 and 2; patient's initial lab investigations along with respective normal values. ALP: Alkaline phosphatase, ALT: Alanine transaminase, AST: Aspartate transaminase, LDH: Lactate dehydrogenase, WBC: White blood cell.

Lab Investigations	Normal Values	Case 1. Initial Lab Findings (On Admission)	Case 2. Initial Lab Findings (On Admission)
ALT (U/L)	10-40	220	131
AST (U/L)	10-40	274	124
ALP (U/L)	35-100	155	173
LDH (U/L)	100-190	809	-
Amylase (U/L)	60-160	191	56
WBC (x10³/ul)	4.40-10.50	17 340	15.6
Neutrophils (x10³/ul)	1.40-8.30	14 370	12.9
Hemoglobin (g/dL)	12.6-17.3	12.7	11.9
Hematocrit (%)	37-45	38.50	33.7
Platelets (x10³/ul)	165-450	204	298
Potassium (mEq/L)	3.5-5.10	5.83	5.43
Lactate (mmol/L)	0.4-2.2	5	-
Urea (mg/dl)	10-50	-	69
Creatinine (mg/dl)	0.50-0.90	-	0.96
Glucose (mg/dL)	70-99	172	171

The patient was immediately transferred to the operating room for a cesarean surgery and an exploratory laparotomy. First, the obstetrics team performed the cesarean section with a midline infraumbilical incision, and a live female infant weighing 1796 grams was delivered with an omphalocele. The surgical team found a ruptured hepatic hematoma affecting the right lobe and part of the left lobe with a hemoperitoneum of approximately 1000 mL. Hemorrhage was managed by liver packaging and ligation of the round ligament and the falciform ligament. It was planned to operate on the fourth day to remove the packaging. A subhepatic drainage was placed, and the abdominal wall was closed. The patient was transferred to the intensive care unit (ICU) where she received 5 units of packed red blood cells, mechanical ventilation, and ionotropic agents. The histopathological result of the placenta demonstrated a mature placenta with microcalcifications, intervillous hemorrhage, vascular congestion, and edema. On the third day in the ICU, the laboratory studies showed no anemia and elevated hepatic enzymes. On the fourth day, the patient was operated again to remove the surgical pads from the previous packaging and aspiration of 600 mL of remaining hemoperitoneum. The patient was discharged on the seventh day with stable vital signs, normalizing hepatic enzymes ALT, 285 U/L; AST, 65 U/L; normal values: ≤ 35 U/L for ALT and ≤ 35 U/L for AST; and mild anemia. The newborn died on the fifth day due to respiratory failure.

Case 2

A 37-year-old woman in her 34 weeks and one day of pregnancy based on the last menstrual period, with a history of two previous cesarean sections, presented to the emergency room with five hours of right hypochondrial pain radiating to the ipsilateral lumbar region. She also experienced vomiting once, with evidence of elevated blood pressure (165/115 mmHg) on two separate occasions within 15 minutes, and proteinuria (protein/creatinine ratio: 0.47). Labetalol and magnesium sulfate treatments were initiated. On physical examination, she exhibited tenderness in the right hypochondrium, a positive Murphy's sign, and a pregnant uterus with fetal movements. A genital examination revealed a closed cervix without vaginal bleeding or discharge.

Obstetric ultrasound showed a single cephalic fetus with a right-sided dorsal presentation, a fetal heart rate of 115 bpm, a posterior placenta, an amniotic fluid index of 6.68 cm, and an estimated fetal weight of 2095 grams. Doppler findings indicated abnormal blood flow in the fetal middle cerebral, umbilical, and uterine arteries.

Upper abdominal ultrasound reported an enlarged liver with normal contours, echogenic parenchyma suggestive of fat infiltration, and heterogeneous echogenicity in the right lobe. The gallbladder was distended with thick walls, containing a 0.9 cm stone. Free fluid was observed around the liver, right kidney, and both flanks.

Laboratory parameters at the presentation are mentioned in Table 1. Laboratory tests indicated increased levels of urea, creatinine, transaminases, and alkaline phosphatase. Blood counts showed leukocytosis with neutrophilia, and electrolyte abnormalities included hyponatremia, hyperkalemia, and decreased serum chloride.

**Figure 2:**
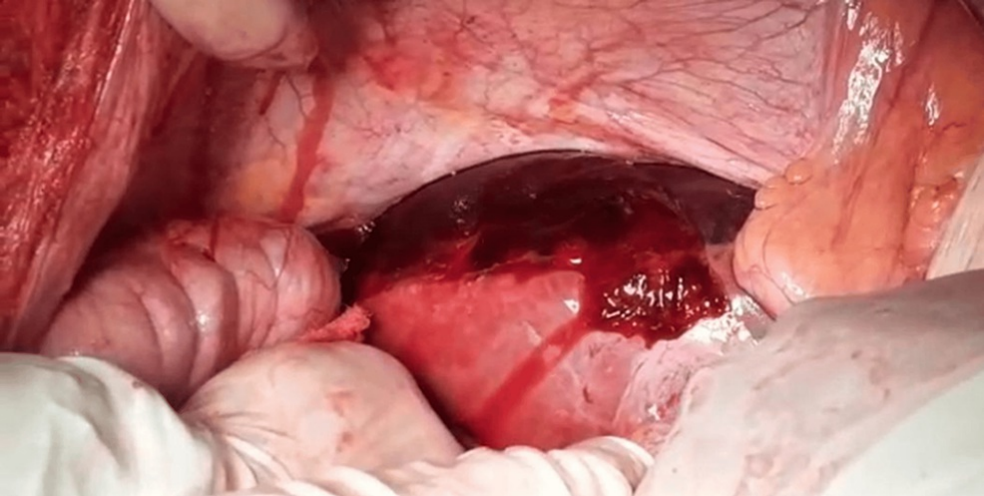
Abdominal exploration reveals abundant clots adhered to the hepatic surface.

During hospitalization, fetal lung maturation was completed with betamethasone two doses of 12 mg intramuscularly 24 hours apart. The patient's abdominal pain increased, and sustained fetal bradycardia (average of 100 bpm in 20 minutes), prompting urgent transfer to the operating room for a cesarean section through midline infraumbilical incision. Intraoperatively, hemoperitoneum was noted, and a Kerr-type hysterotomy was performed. A live female fetus was delivered with clear amniotic fluid, followed by normal placental delivery and a two-layer uterine repair. Uterine atony was identified, which did not improve with doses of 40 IU of oxytocin and 600 mcg of misoprostol. Consequently, a decision was made to perform a modified B-Lynch suture. Exploration revealed extensive clots in the liver, prompting consultation with general surgery for hepatic packing (Figure 2).

Seven compresses were used for hepatic packing, followed by the closure of the abdominal cavity in two layers. The patient was transferred to the intensive care unit. Postoperative hemoglobin level (9.1 mg/dL) led to the transfusion of three units of packed red blood cells. Hepatic packing was removed without complications after 72 hours. Subsequent laboratory results showed normal renal function, decreased transaminases (AST: 32 U/L, ALT: 72 U/L), reduced leukocytic response, moderate anemia (hemoglobin: 10.0 g/dL, hematocrit: 28.6%), normal platelets (363 x10³/μL), and normal electrolytes.

Nine days after admission, with a clinically favorable course, the decision was made for discharge. Both the newborn and mother were discharged in good clinical condition.

## Discussion

Preeclampsia is a multisystem disorder affecting approximately 2-5% of pregnant women, being a leading cause of maternal and perinatal morbidity and mortality [10]. Spontaneous hepatic rupture as a complication of pregnancy is extremely rare, but associated with high mortality, often linked to preeclampsia, eclampsia, and more frequently with HELLP syndrome [11]. HELLP syndrome is clinically associated with preeclampsia, characterized by hemolysis, elevated liver enzymes, and decreased platelets [12].

Complications of HELLP syndrome include disseminated intravascular coagulation (20%), placental abruption (16%), acute renal failure (7%), acute pulmonary edema (6%), subcapsular hepatic hematoma (1.8%), and hepatic rupture (0.9%) [13-15].

In pregnant women diagnosed with HELLP syndrome, a high index of suspicion is necessary for abdominal pain without an apparent cause and early signs of hypovolemic shock. Additional imaging studies (ultrasound, CT scan, MRI) and laboratory tests are required to identify hepatic lesions or intra-abdominal bleeding early on [16]. Aminotransferases may only moderately elevate in the context of hepatic bleeding, with a poor correlation between the magnitude of histological hepatic abnormalities and laboratory abnormalities [13,17,18]. Clinically, hepatic rupture presents with right hypochondrial or epigastric abdominal pain, radiating to the right shoulder, accompanied by nausea, vomiting, and hemodynamic alterations. Hepatic rupture can occur before, during, or after delivery [19]. The diagnosis remains a challenging task because of the unspecific physical symptoms and signs. In fact, laboratory findings indirectly contribute to the final diagnosis and do not predict the severity of each case.

The pathophysiology of hepatic rupture remains under investigation, with a proposed mechanism involving intravascular and periportal fibrin deposits leading to sinusoidal obstruction, intrahepatic vascular congestion, and hepatic ischemia or necrosis [20].

HELLP syndrome complicated by subcapsular hematoma or hepatic rupture is considered a medical emergency, and immediate termination of pregnancy via cesarean section is standard, with hepatic surgical management if required. Hepatic packing for bleeding control, fibrin sealant application, direct suturing of damaged hepatic tissue, ligation or embolization of the hepatic artery, hepatic lobectomy, orthotopic liver transplantation, or a combination of these procedures may be necessary [21-23]. 

Two cases of hepatic rupture related to hypertensive disorders of pregnancy are presented, with one newborn death due to complications related to prematurity and omphalocele. Hepatic rupture in severe preeclampsia poses serious problems, increasing the risk of maternal and fetal mortality. Both patients required surgical management for hepatic rupture, including hepatic packing for hemorrhage control, hemodynamic management, and packed red blood cell transfusions for hemorrhage. Subsequently, they required comprehensive care in the intensive care unit, remaining hospitalized for several days until discharge. In both cases, mothers were discharged with good clinical evolution, and only one newborn survived, emphasizing the need for aggressive and comprehensive management involving obstetrics, radiology, intensive care, general surgery, and hematology, among other services, for improved outcomes. Therefore, these pathologies must be addressed in high-level centers with multidisciplinary support.

Clinical improvement and recovery in patients who have experienced HELLP syndrome usually occur between days 4 and 11, with normalization of hepatic laboratory parameters on the fourth postpartum day. However, a certain group of patients may experience delayed resolution [24-26]. Long-term follow-up of these patients, especially those with renal, hepatic, or pulmonary complications, is essential. To manage subsequent pregnancies in patients who have a history of preeclampsia or HELLP syndrome, it is important to monitor for signs or symptoms of related complications early and to take preventive measures like low-dose aspirin [27].

The rates of recurrence of subcapsular hematomas and hepatic ruptures in subsequent pregnancies are not well known [18]. There is a need for frequent and thorough evaluation in patients with a history of HELLP syndrome in previous pregnancies.

## Conclusions

HELLP (hemolysis, elevated liver enzymes, and low platelets) syndrome is a rare complication that significantly impacts maternal and fetal morbidity, necessitating prompt attention through an assertive multidisciplinary approach. In this context, hepatic rupture emerges as an infrequent but critical complication in HELLP syndrome, demanding immediate and precise management due to its potential to jeopardize the survival of both the mother and the fetus.

In patients with hepatic rupture, clinical improvement may take several weeks, and laboratory values may take several days, on average up to four, to normalize, but it could take even longer. Therefore, extended monitoring is necessary until these values return to normal.

The recurrence risk of complications such as HELLP syndrome, hepatic rupture, or hematoma in subsequent pregnancies remains not fully understood, necessitating further research. Enhanced surveillance and heightened suspicion are imperative in subsequent pregnancies when clinical or laboratory abnormalities are present.
